# An air quality index prediction model based on CNN-ILSTM

**DOI:** 10.1038/s41598-022-12355-6

**Published:** 2022-05-19

**Authors:** Jingyang Wang, Xiaolei Li, Lukai Jin, Jiazheng Li, Qiuhong Sun, Haiyao Wang

**Affiliations:** 1 School of Information Science and Engineering, Hebei University of Science and Technology, Shijiazhuang, 050018 China; 2 School of Ocean Mechatronics, Xiamen Ocean Vocational College, Xiamen, 361100 China

**Keywords:** Environmental sciences, Engineering

## Abstract

Air quality index (AQI) is an essential measure of air pollution evaluation, which describes the air pollution degree and its impact on health, so the accurate prediction of AQI is significant. This paper presents an AQI prediction model based on Convolution Neural Networks (CNN) and Improved Long Short-Term Memory (ILSTM), named CNN-ILSTM. ILSTM deletes the output gate in LSTM and improves its input gate and forget gate, and introduces a Conversion Information Module (CIM) to prevent supersaturation in the learning process. ILSTM realizes efficient learning of historical data, improves prediction accuracy, and reduces the training time. CNN extracts the eigenvalues of input data effectively. This paper uses air quality data from 00:00 on January 1, 2017, to 23:00 on June 30, 2021, in Shijiazhuang City, Hebei Province, China, as experimental data sets, and compares this model with eight prediction models: SVR, RFR, MLP, LSTM, GRU, ILSTM, CNN-LSTM, and CNN-GRU to prove the validity and accuracy of CNN-ILSTM prediction model. The experimental results show the MAE of CNN-ILSTM is 8.4134, MSE is 202.1923, R^2^ is 0.9601, and the training time is 85.3 s. In this experiment, the performance of this model performs better than other models.

## Introduction

The problem of urban air pollution is becoming increasingly severe, which has become an important factor hindering the sustainable development of Chinese cities and the construction of ecological civilization^1,2^. Air quality affects people's lives, production, and health. AQI is an essential basis for measuring air quality.

The AQI is divided into six levels^3,4^. The first level of the AQI is between 0 and 50, and there is little air pollution. The second level is between 51 and 100, and the pollutants may have a weak effect on the health of a very small number of exceptionally sensitive people. The third level is between 101 and 150, and the symptoms of susceptible people are mildly aggravated, and healthy people have irritating symptoms. The fourth level is between 151 and 200, which means that the heart and respiratory system of healthy people are affected. The fifth level is between 201 and 300, patients with heart and lung disease significantly increase symptoms. AQI above 300 is the sixth level, healthy people reduce exercise tolerance, have obvious and strong symptoms, and certain diseases appear in advance^5,6^.

Therefore, it is crucial to accurately predict the changes of AQI in a short time in the future by using historical observation data^7–9^. In recent years, China has been increasing investment in the construction of air pollution indicators monitoring. In 2010, China established an active monitoring system of air pollution indicators in key cities. By the end of 2015, the automatic monitoring of air quality indicators has covered more than 300 cities. Although China’s the monitoring system is good, the problems of high cost, high energy consumption, and low-level efficiency appear sometimes. It is necessary to build a more reasonable prediction model to enhance the effectiveness and accuracy of air quality monitoring and reduce production costs.

The traditional fully connected method in a neural network has many problems, such as the inability to utilize time-series information in data and too many parameters. With the proposal of Recurrent Neural Network (RNN), RNN has achieved a significant breakthrough in time series analysis, language model, speech recognition, and machine translation^10–14^. Because of the traditional RNN's long-term dependence on data, the issues of “gradient explosion” and “gradient disappearance” often appear in model training when the amount of data is large^15–19^. The emergence of LSTM can alleviate the issues of “gradient explosion” and “gradient disappearance” in RNN's training process^20,21^. It uses gated technology to determine the participation degree of historical data.

The time series prediction based on LSTM has too many parameters, so the calculation of the model is too complex, resulting in too long a training time^22–24^. Therefore, ILSTM proposed in this paper deletes the output gate in LSTM, improves the input gate and forget gate in LSTM, and introduces a CIM to prevent supersaturation in the learning process. ILSTM aims to reduce the model's training time and improve model prediction accuracy on the premise that the model can alleviate the issues of “gradient explosion” and “gradient disappearance” of the RNN. ILSTM realizes the effective control of "forget gate" and "input gate" to time series data through simplified calculation. This paper presents an AQI prediction model based on CNN-ILSTM. In the model, CNN can achieve eigenvalues extraction well and make up for the insufficient feature extraction and learning of ILSTM. The experiment introduces eight different models as baseline models to verify this model's effectiveness. The prediction results are evaluated as a whole by using Mean Absolute Error (MAE), Mean Squared Error (MSE), R Squared (R^2^), and training time. The experiment shows that the MAE, MSE, R^2^, and training time of CNN-ILSTM are 8.4134,202.1923, 0.9601, and 85.3 s, respectively. Comparative experiment shows that the CNN-ILSTM prediction model is superior to other models in the overall evaluation. To sum up, the contributions of this paper are as follows:

1. Through the research of RNN and LSTM and time-series data, this paper presents an improved LSTM network, ILSTM, which deletes the output gate of LSTM, improves the input gate and forget gate of LSTM, and introduces a CIM to prevent supersaturation in the learning process.
2. Compared with LSTM, ILSTM proposed in this paper has fewer parameters, lower computational complexity, and less training time on the premise of ensuring the prediction accuracy of AQI.
3. This paper presents an AQI prediction model based on CNN-ILSTM. The introduction of CNN can well extract eigenvalues of input data. Through comparative experiment, the combination of CNN and ILSTM can improve the accuracy of AQI prediction. And compared with the other eight prediction models, the AQI prediction result based on CNN-ILSTM performs better.

## Related work

Traditional regression models for time series prediction include Random Forest Regression (RFR), Support Vector Regression (SVR), and Multi-Layer Perceptron (MLP). Ganesh et al. used SVR to forecast the AQI of Delhi and Houston^25^. However, because of the unstable characteristics of AQI data, it was difficult for SVR to achieve a high fitting degree. Zhang et al. proposed the RFR based on Spark clustering for air quality prediction^26^, but for the prediction of nonlinear air quality data, the RFR had the risk of over-fitting. Duro et al. used MLP to forecast the concentration of PM10, and O_3_ in industrial areas^27^. However, for many non-stationary time series data, the traditional MLP prediction model had the problem of low prediction fitting degree.

RNN significantly improved the fitting degree of time series prediction. Compared with standard neural networks, the calculation results of RNN's every hidden layer were related to the current input and the last hidden layer's result. By this method, the calculation result of RNN had the characteristic of remembering the previous results. For example, Wang used RNN to predict air quality^28^. Because of RNN's long-term dependence on data, the issues of “gradient explosion” and “gradient disappearance” will appear during model training^29^.

The gated technology has alleviated the issues of “gradient explosion” and “gradient disappearance” caused by the RNN’s long-term dependence on data to a great extent. For example, Ysc et al. used LSTM to forecast changes in air pollutants^30^. Because of the single prediction model, the extraction of eigenvalues was often insufficient, making it difficult to achieve high precision prediction. Dsa et al. proposed air quality prediction based on LSTM^31^. Alhirmizy et al. used LSTM to forecast the multivariate time series of air pollution in Madrid, Spain^32^. To improve the prediction accuracy, Dsa^31^ and Alhirmizy^32^ used diversified data with increased data volume, which would lead to the problem of increasing model training time. Cwa et al. proposed an AQI prediction model based on CNN-LSTM, which combines CNN and LSTM to improve the ability for extracting features and integrating air quality data, thus improving the prediction accuracy^33^. Zhu et al. used the CNN-LSTM hybrid model in the process of PM2.5 prediction^34^. Cwa and Zhu et al. used CNN to make up for the problem of insufficient feature extraction of LSTM to a great extent, but LSTM itself often takes a long time to train because of many parameters.

## Models

### CNN

**Figure 1:**
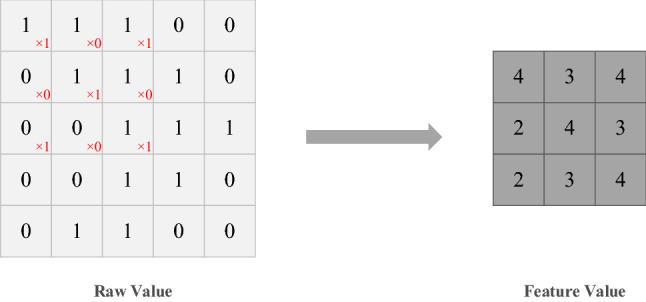
CNN convolutional layer feature extraction process.

Compared with the traditional neural network model, CNN has some unique advantages. For example, with the increase of hidden layers and nodes of the neural network, traditional neural network W weight parameters and B biases parameters will gradually increase, so the amount of calculation will also gradually increase. But CNN realizes parameter sharing, so the amount of calculation is greatly reduced^35,36^, as shown in Fig. 1. CNN can handle more complex data environments and problems with unclear data background and unclear inference rules, and allow the sample to have larger defects and distortions^37–40^. CNN can also well realize feature extraction of local signals, and the combination of CNN, RNN, and LSTM has been widely used in feature extraction of time series data^41–43^. Therefore, CNN can effectively extract features from non-linear and unstable air quality data.

### ILSTM

#### Model structure

**Figure 2:**
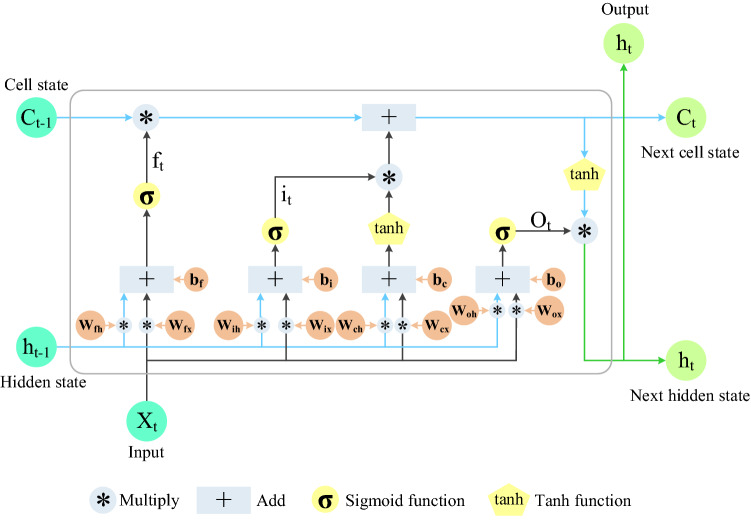
LSTM structure.

**Figure 3:**
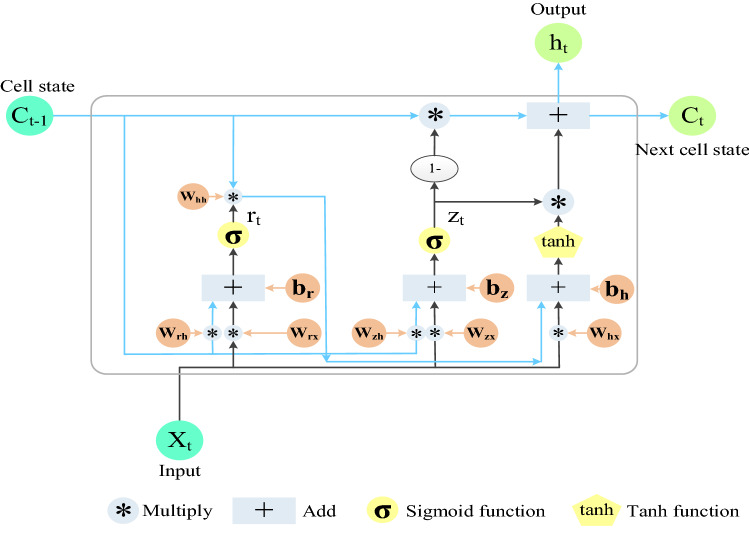
GRU structure.

**Figure 4:**
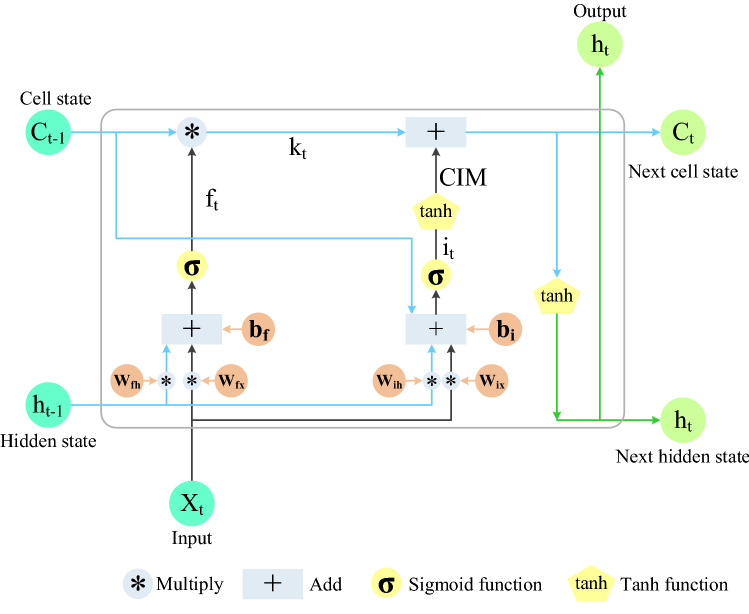
ILSTM structure.

LSTM has excellent advantages in mining long-term dependence relationships of sequence data. Figure 2 shows three types of gates: forget gate, input gate, and output gate, respectively. A gate can be regarded as a full connection layer, and LSTM stores and updates information by these gates^44,45^. Gated Recurrent Unit (GRU) has only two gates. GRU combines the input gate and forget gate in LSTM into one, which is called the update gate^46^, as shown in Fig. 3. Based on the gated technology, the ILSTM model proposed in this paper consists of input gate and forget gate, as shown in Fig. 4.

Compared with LSTM, ILSTM deletes the output gate. Compared with GRU, ILSTM structure is simpler. The parameters of LSTM, GRU, and ILSTM are shown in Table 1. Compared with LSTM, ILSTM reduces weight parameters from 8 to 4 and bias parameters from 4 to 2. Compared with GRU, ILSTM reduces weight parameters from 6 to 4 and bias parameters from 3 to 2.

**Table 1:** The parameters of LSTM and ILSTM model.

LSTM	GRU	ILSTM
3 gates	2 gates	2 gates
8 weight parameters	6 weight parameters	4 weight parameters
4 bias parameters	3 bias parameters	2 bias parameters

In terms of algorithm, ILSTM adds the cell state ${\mathrm{c}}_{\mathrm{t}-1}$ of the previous moment to the algorithm of the forget gate to generate the mainline forgetting ${\mathrm{k}}_{\mathrm{t}}$, which affects the data retention degree at the current time. In addition, when updating the cell state ${\mathrm{c}}_{\mathrm{t}}$ of the current moment, the CIM is introduced to prevent supersaturation in the learning process.

The forget gate of the ILSTM ${\mathrm{f}}_{\mathrm{t}}$ is a crucial component of ILSTM unit, which can control what information should be retained and what information should be forgotten. $\sigma (\mathrm{x})$ is a Sigmoid function, as shown in formula (1). ${\mathrm{x}}_{\mathrm{t}}$ is the input data of the t-th time step. ${\mathrm{h}}_{\mathrm{t}-1}$ is the hidden layer of the previous time step t − 1. ${\mathrm{W}}_{\mathrm{fh}}$ is the weight of ${\mathrm{h}}_{\mathrm{t}-1}$ of forget gate, and ${\mathrm{W}}_{\mathrm{fx}}$ is the weight of ${\mathrm{x}}_{\mathrm{t}}$. ${\mathrm{b}}_{\mathrm{f}}$ is the bias of forget gate, as shown in formula (2).

1 $$\sigma \left(\mathrm{x}\right)=\frac{1}{1+{e}^{-x}},$$

2 $${\mathrm{f}}_{\mathrm{t}}=\sigma \left({\mathrm{W}}_{\mathrm{fh}}\cdot {\mathrm{h}}_{\mathrm{t}-1}+{{\mathrm{W}}_{\mathrm{fx}}\cdot \mathrm{x}}_{\mathrm{t}}+{\mathrm{b}}_{\mathrm{f}}\right).$$

Mainline forgetting ${\mathrm{k}}_{\mathrm{t}}$ is calculated by cell state ${\mathrm{c}}_{\mathrm{t}-1}$ and ${\mathrm{f}}_{\mathrm{t}}$. Mainline forgetting represents the influence of information on current cell state ${\mathrm{c}}_{\mathrm{t}}$, where ${\mathrm{c}}_{\mathrm{t}-1}$ is cell state information from the beginning to the previous moment, as shown in formula (3).

3 $${\mathrm{k}}_{\mathrm{t}}={\mathrm{f}}_{\mathrm{t}}\times {\mathrm{c}}_{\mathrm{t}-1}.$$

The input gate ${\mathrm{i}}_{\mathrm{t}}$ controls how much of the current input data ${\mathrm{x}}_{\mathrm{t}}$ flows into the memory cell, that is, how much can be saved to ${\mathrm{c}}_{\mathrm{t}}$. Compared with the input gate of LSTM, ILSTM adds ${\mathrm{c}}_{\mathrm{t}-1}$ to the input gate algorithm, that is, the cell state information up to the previous moment. The introduction of ${\mathrm{c}}_{\mathrm{t}-1}$ makes the input gate of the model have a memory effect on the retention of data at the current time, as shown in formula (4), ${\mathrm{W}}_{\mathrm{ih}}$ and ${\mathrm{W}}_{\mathrm{ix}}$ are the weights of the input gate’s ${\mathrm{h}}_{\mathrm{t}-1}$ and ${\mathrm{x}}_{\mathrm{t}}$, respectively, and ${\mathrm{b}}_{\mathrm{i}}$ is the bias of the input gate.

4 $${\mathrm{i}}_{\mathrm{t}}=\sigma \left({\mathrm{W}}_{\mathrm{ih}}\cdot {\mathrm{h}}_{\mathrm{t}-1}+{{\mathrm{W}}_{\mathrm{ix}}\cdot \mathrm{x}}_{\mathrm{t}}+{\mathrm{c}}_{\mathrm{t}-1}+{\mathrm{b}}_{\mathrm{i}}\right).$$

**Figure 5:**
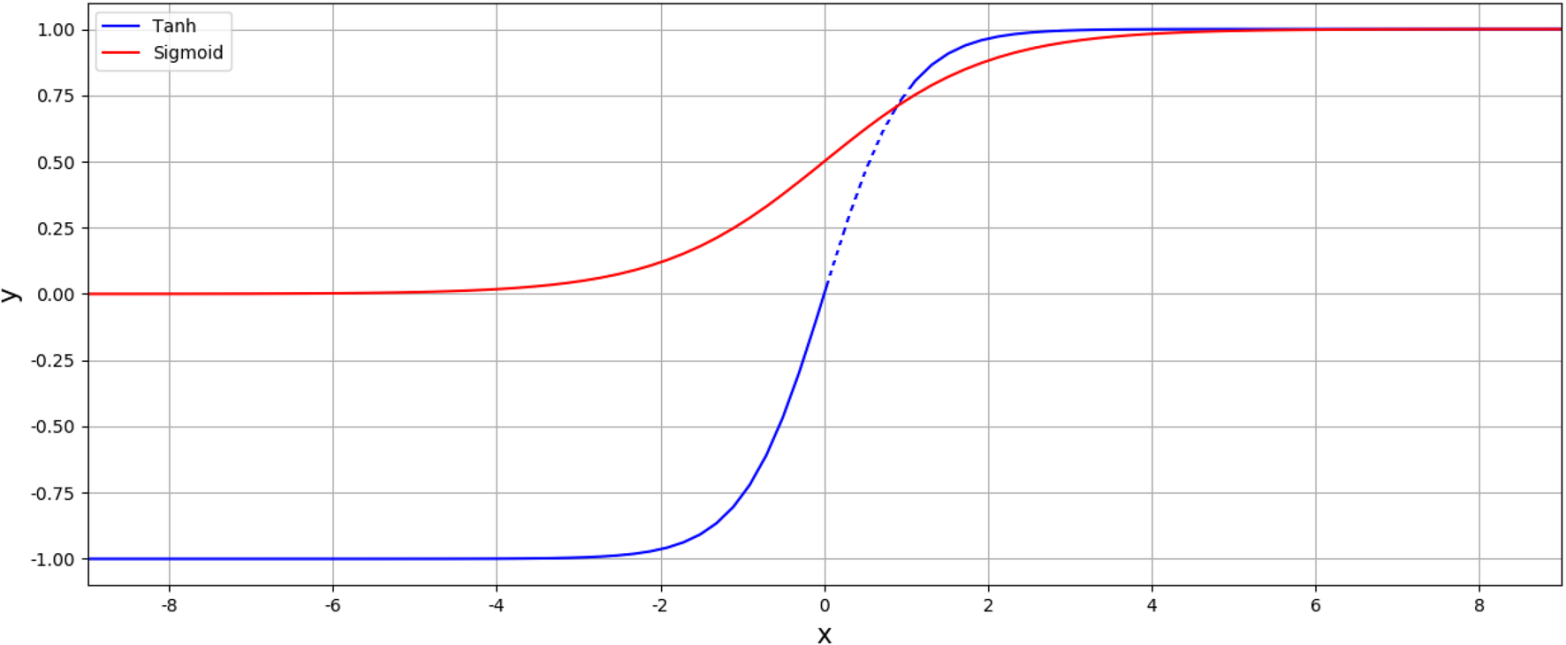
Tanh and Sigmoid function.

Due to the characteristics of the Sigmoid activation function, when the value of x is outside − 3 and 3, the value of the Sigmoid activation function will fall into a supersaturation interval. Therefore, in formula (4), when the input data enters the input gate's supersaturation, the value does not change significantly, decreasing learning sensitivity. A CIM is introduced into ILSTM model to prevent this phenomenon, As shown in formula (5). The Sigmoid function value (ranging from 0 to 1) calculated by the above formula is taken as the input of Tanh. Tanh and Sigmoid function as shown in Fig. 5.

The value of $\mathrm{tanh}({\mathrm{i}}_{\mathrm{t}})$ will be between [0, 0.762), as shown in the dotted line part by the tanh function in Fig. 5, so the obtained value will be more uniform and significant. Therefore, the value output by the CIM will greatly reduce the supersaturation degree, and the significant difference makes the model calculation more recognizable, thereby making the model learning more sensitive.

5 $$\mathrm{CIM}=\mathrm{tanh}\left({\mathrm{i}}_{\mathrm{t}}\right).$$

Formula (6) shows that ${\mathrm{c}}_{\mathrm{t}}$ is the information kept from the beginning to the present.

6 $${\mathrm{c}}_{\mathrm{t}}={\mathrm{k}}_{\mathrm{t}}+\mathrm{CIM}.$$

${\mathrm{h}}_{\mathrm{t}}$ indicates the information preserved at the current time. ${\mathrm{c}}_{\mathrm{t}}$ controls how much information can be kept through tanh function, as shown in formula (7).

7 $${\mathrm{h}}_{\mathrm{t}}=\mathrm{tanh}\left({\mathrm{c}}_{\mathrm{t}}\right).$$

#### Formula derivation of ILSTM

ILSTM is proposed to improve the model's prediction accuracy and reduce the model's training time on the premise that the model can alleviate the issues of “gradient explosion” and “gradient disappearance” of the RNN. Input gate and forget gate use two parameter matrices $\left[{\mathrm{W}}_{\mathrm{fh}},{\mathrm{W}}_{\mathrm{fx}}\right]$ and $\left[{\mathrm{W}}_{\mathrm{ih}},{\mathrm{W}}_{\mathrm{ix}}\right]$. Record ${\mathrm{W}}_{\mathrm{f}}=\left[{\mathrm{W}}_{\mathrm{fh}},{\mathrm{W}}_{\mathrm{fx}}\right]$, ${\mathrm{W}}_{\mathrm{i}}=\left[{\mathrm{W}}_{\mathrm{ih}},{\mathrm{W}}_{\mathrm{ix}}\right]$,and $\mathrm{W}=[{\mathrm{W}}_{\mathrm{f}},{\mathrm{W}}_{\mathrm{i}}]$. The ${\mathrm{L}}_{\mathrm{t}}$ function of $\mathrm{W}$ is the loss corresponding to ${\mathrm{h}}_{\mathrm{t}}$. $\mathrm{L}$ is the total loss. As for the derivative of $\mathrm{W}$ of $\mathrm{L}$, as shown in formula (8):

8 $$ \frac{{\partial {\text{L}}}}{{\partial {\text{W}}}} = \mathop \sum \limits_{{{\text{t}} = 1}}^{{\text{T}}} \frac{{\partial {\text{L}}_{{\text{t}}} }}{{\partial {\text{W}}}}. $$

The RNN updates the W parameter by formula (9):

9 $$\mathrm{W}=\mathrm{W}-\frac{\partial {\mathrm{L}}_{\mathrm{t}}}{\partial \mathrm{W}},$$

where $\frac{\partial{\text{L}}_{\text{t}}}{\partial \mathrm{W}}$ can be written as formula (10):

10 $$\frac{\partial {\mathrm{L}}_{\mathrm{k}}}{\partial \mathrm{W}}=\frac{\partial {\mathrm{L}}_{\mathrm{k}}}{\partial {\mathrm{h}}_{\mathrm{k}}}\frac{\partial {\mathrm{h}}_{\mathrm{k}}}{\partial {\mathrm{c}}_{\mathrm{k}}}\frac{\partial {\mathrm{c}}_{\mathrm{k}}}{\partial {\mathrm{c}}_{\mathrm{k}-1}}\dots \frac{\partial {\mathrm{c}}_{2}}{\partial {\mathrm{c}}_{1}}\frac{\partial {\mathrm{c}}_{1}}{\partial \mathrm{W}}.$$

Formula (10) can be simplified to formula (11):

11 $$ \frac{{\partial {\text{L}}_{{\text{k}}} }}{{\partial {\text{W}}}} = \frac{{\partial {\text{L}}_{{\text{k}}} }}{{\partial {\text{h}}_{{\text{k}}} }}\frac{{\partial {\text{h}}_{{\text{k}}} }}{{\partial {\text{c}}_{{\text{k}}} }} \ldots \frac{{\partial {\text{c}}_{2} }}{{\partial {\text{c}}_{1} }}\frac{{\partial {\text{c}}_{1} }}{{\partial {\text{W}}}} = \frac{{\partial {\text{L}}_{{\text{k}}} }}{{\partial {\text{h}}_{{\text{k}}} }}\frac{{\partial {\text{h}}_{{\text{k}}} }}{{\partial {\text{c}}_{{\text{k}}} }}\left( {\mathop \prod \limits_{{{\text{t}} = 2}}^{{\text{k}}} \frac{{\partial {\text{c}}_{{\text{t}}} }}{{\partial {\text{c}}_{{{\text{t}} - 1}} }}} \right)\frac{{\partial {\text{c}}_{1} }}{{\partial {\text{W}}}}, $$

where ${\mathrm{c}}_{\mathrm{t}}$ is shown in formula (12):

12 $${\mathrm{c}}_{\mathrm{t}}={\mathrm{f}}_{\mathrm{t}}\times {\mathrm{c}}_{\mathrm{t}-1}+\mathrm{CIM}.$$

The $\mathrm{CIM}=\mathrm{tanh}\left({\mathrm{i}}_{\mathrm{t}}\right)$, so formula (12) can be written as formula (13):

13 $${\mathrm{c}}_{\mathrm{t}}={\mathrm{f}}_{\mathrm{t}}\times {\mathrm{c}}_{\mathrm{t}-1}+\mathrm{tanh}\left({\mathrm{i}}_{\mathrm{t}}\right).$$

The derivative of ${\mathrm{c}}_{\mathrm{t}}$ can be obtained by formula (14):

14 $$\frac{\partial {\mathrm{c}}_{\mathrm{t}}}{\partial {\mathrm{c}}_{\mathrm{t}-1}}={\mathrm{f}}_{\mathrm{t}}+(1{-\mathrm{tanh}\left({\mathrm{i}}_{\mathrm{t}}\right)}^{2}){(\mathrm{i}}_{\mathrm{t}}){^{\prime}}.$$

Then the total loss can be written as formula (15):

15 $$ \frac{{\partial {\text{L}}_{{\text{k}}} }}{{\partial {\text{W}}}} = \frac{{\partial {\text{L}}_{{\text{k}}} }}{{\partial {\text{h}}_{{\text{k}}} }}\frac{{\partial {\text{h}}_{{\text{k}}} }}{{\partial {\text{c}}_{{\text{k}}} }}\left( {\mathop \prod \limits_{{{\text{t}} = 2}}^{{\text{k}}} \left( {{\text{f}}_{{\text{t}}} + \left( {1 - \tanh \left( {{\text{i}}_{{\text{t}}} } \right)^{2} } \right)({\text{i}}_{{\text{t}}} ){^{\prime}}} \right)} \right)\frac{{\partial {\text{c}}_{1} }}{{\partial {\text{W}}}}. $$

Then record that $\mathrm{x}$ is equal to formula (16):

16 $$\mathrm{x}={\mathrm{W}}_{\mathrm{fh}}\cdot {\mathrm{h}}_{\mathrm{t}-1}+{{\mathrm{W}}_{\mathrm{fx}}\cdot \mathrm{x}}_{\mathrm{t}}+{\mathrm{b}}_{\mathrm{f}}.$$

Then that ${\mathrm{f}}_{\mathrm{t}}$ can be written as formula (17):

17 $${\mathrm{f}}_{\mathrm{t}}=\sigma \left({\mathrm{W}}_{\mathrm{fh}}\cdot {\mathrm{h}}_{\mathrm{t}-1}+{{\mathrm{W}}_{\mathrm{fx}}\cdot \mathrm{x}}_{\mathrm{t}}+{\mathrm{b}}_{\mathrm{f}}\right)=\sigma \left(\mathrm{x}\right).$$

Then record that $\mathrm{y}$ is equal to formula (18):

18 $${\mathrm{y}=\mathrm{W}}_{\mathrm{ih}}\cdot {\mathrm{h}}_{\mathrm{t}-1}+{{\mathrm{W}}_{\mathrm{ix}}\cdot \mathrm{x}}_{\mathrm{t}}+{\mathrm{c}}_{\mathrm{t}-1}+{\mathrm{b}}_{\mathrm{i}}.$$

Then that ${\mathrm{i}}_{\mathrm{t}}$ can be written as formula (19):

19 $${\mathrm{i}}_{\mathrm{t}}=\sigma \left({\mathrm{W}}_{\mathrm{ih}}\cdot {\mathrm{h}}_{\mathrm{t}-1}+{{\mathrm{W}}_{\mathrm{ix}}\cdot \mathrm{x}}_{\mathrm{t}}+{\mathrm{c}}_{\mathrm{t}-1}+{\mathrm{b}}_{\mathrm{i}}\right)=\sigma \left(y\right).$$

Then the formula (15) can be written as formula (20):

20 $$ \frac{{\partial {\text{L}}_{{\text{k}}} }}{{\partial {\text{W}}}} = \frac{{\partial {\text{L}}_{{\text{k}}} }}{{\partial {\text{h}}_{{\text{k}}} }}\frac{{\partial {\text{h}}_{{\text{k}}} }}{{\partial {\text{c}}_{{\text{k}}} }}\left( {\mathop \prod \limits_{{{\text{t}} = 2}}^{{\text{k}}} \left( {\sigma \left( {\text{x}} \right) + \left( {1 - \tanh \left( {\sigma \left( y \right){ }} \right)^{2} } \right)\sigma^{\prime}\left( y \right)} \right)} \right)\frac{{\partial {\text{c}}_{1} }}{{\partial {\text{W}}}}. $$

Then record that $\mathrm{z}(\mathrm{x},\mathrm{y})$ is equal to formula (21):

21 $$\mathrm{z}(\mathrm{x},\mathrm{y})= \sigma \left(\mathrm{x}\right)+{(1-\mathrm{tanh}\left(\sigma \left(\mathrm{y}\right)\right)}^{2})\sigma {^{\prime}}(\mathrm{y}).$$

Then the formula (20) can be written as formula (22):

22 $$ \frac{{\partial {\text{L}}_{{\text{k}}} }}{{\partial {\text{W}}}} = \frac{{\partial {\text{L}}_{{\text{k}}} }}{{\partial {\text{h}}_{{\text{k}}} }}\frac{{\partial {\text{h}}_{{\text{k}}} }}{{\partial {\text{c}}_{{\text{k}}} }}\left( {\mathop \prod \limits_{{{\text{t}} = 2}}^{{\text{k}}} \left( {{\text{z}}\left( {{\text{x}},{\text{y}}} \right)} \right)} \right)\frac{{\partial {\text{c}}_{1} }}{{\partial {\text{W}}}}. $$

As shown in formula (22), the gradient of the function is $\frac{\partial {\mathrm{L}}_{\mathrm{k}}}{\partial {\mathrm{h}}_{\mathrm{k}}}\frac{\partial {\mathrm{h}}_{\mathrm{k}}}{\partial {\mathrm{c}}_{\mathrm{k}}}\left(\prod_{\mathrm{t}=2}^{\mathrm{k}}\left(\mathrm{z}(\mathrm{x},\mathrm{y})\right)\right)\frac{\partial {\mathrm{c}}_{1}}{\partial \mathrm{W}}$. When $\mathrm{z}(\mathrm{x},\mathrm{y})$ is greater than 1, the gradient may be too large with the increase of data amount. When $\mathrm{z}(\mathrm{x},\mathrm{y})$ is too small, the gradient disappears easily.

**Figure 6:**
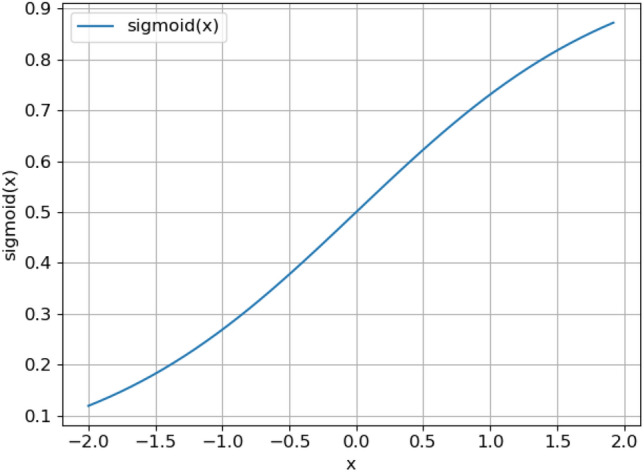
$$\sigma \left(\mathrm{x}\right)$$ Function.

**Figure 7:**
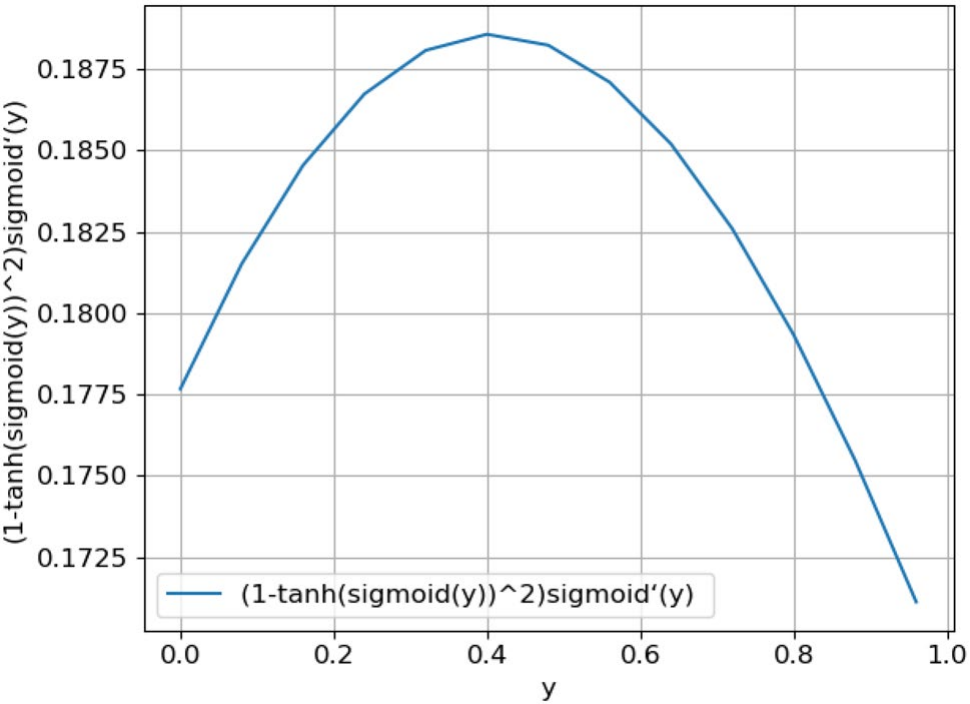
$${(1-\mathrm{tanh}\left(\sigma (\mathrm{y})\right)}^{2})\sigma {^{\prime}}(\mathrm{y})$$ Function.

**Figure 8:**
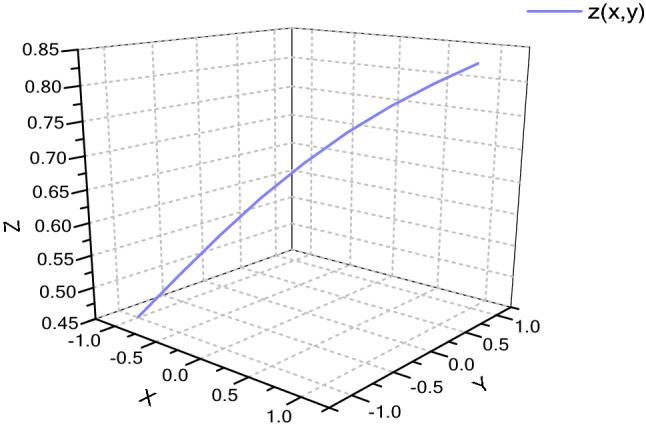
z (x, y) function.

In this model, the $\sigma \left(\mathrm{x}\right)$ function is shown in Fig. 6, and the ${(1-\mathrm{tanh}\left(\sigma (\mathrm{y})\right)}^{2})\sigma {^{\prime}}(\mathrm{y}$) function is shown in Fig. 7. $\sigma (\mathrm{x})$'s range is [0,1] and $\sigma (\mathrm{y})$ 's range is [0, 1], so the function range of ${(1-\mathrm{tanh}\left(\sigma (\mathrm{y})\right)}^{2})\sigma {^{\prime}}(\mathrm{y})$ is (0.1720, 0.1880). Function gradient $\mathrm{z}(\mathrm{x},\mathrm{y})$ is shown in Fig. 8. It can be seen from the figure that the value range of function gradient $\mathrm{z}(\mathrm{x},\mathrm{y})$ will be more reasonable. Therefore, this model can alleviate the problems of "gradient disappearance" and "gradient explosion" to a great extent.

### CNN-ILSTM

**Figure 9:**
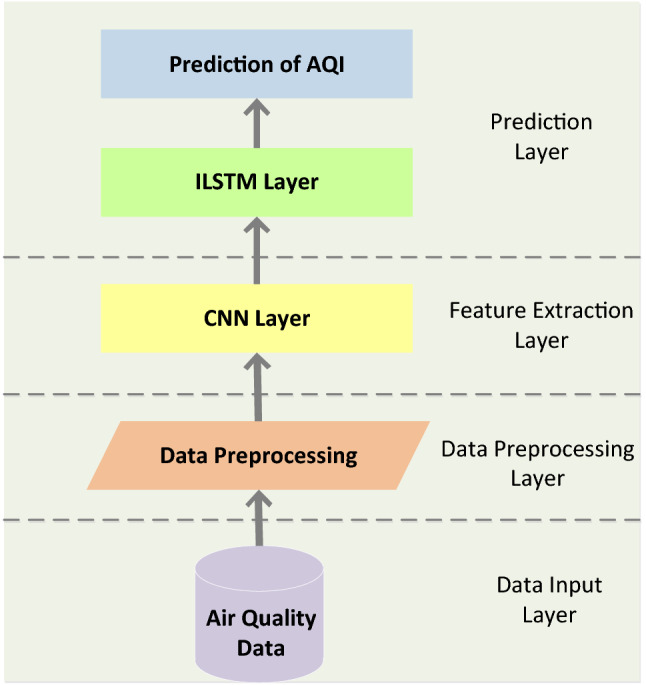
CNN-ILSTM structure overview.

The structure of CNN-ILSTM is shown in Fig. 9. The CNN-ILSTM model is generally divided into four parts. The first layer is the data input layer. This paper takes AQI as the research object and air quality data as the model input. The second layer is the data preprocessing layer. To ensure the reliability of the prediction results and improve the accuracy of the prediction results, it is necessary to conduct standardized processing of the original data, three-dimensional time series construction and other pre-processing operations. The third layer is the feature extraction layer, which realizes feature extraction of air quality data by taking advantage of CNN's significant advantages in feature extraction. The last layer is the prediction layer. Through the optimized ILSTM model, the prediction of AQI is realized and the prediction accuracy is improved.

**Figure 10:**
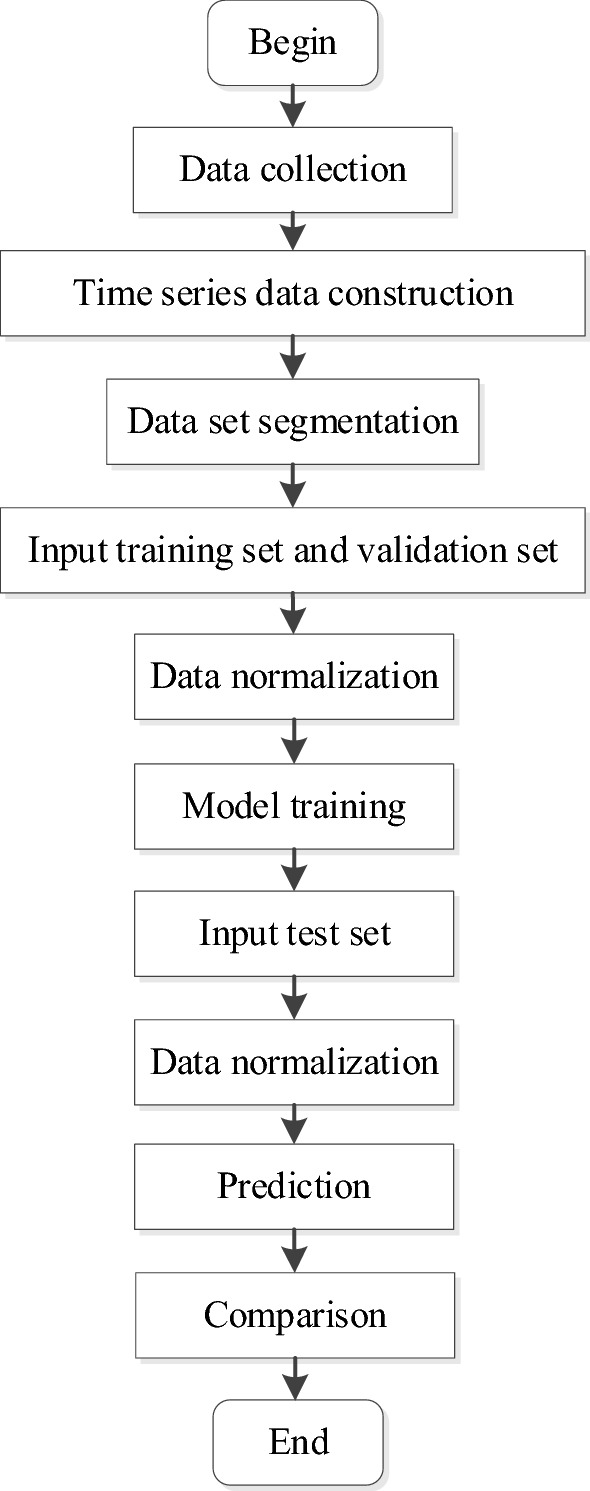
CNN-ILSTM network AQI prediction process.

The AQI prediction process of CNN-ILSTM is shown in Fig. 10.

## Experiment

### Experiment environment

This experiment runs on Windows 10 operating system, and the hardware device is Intel(R) Core(TM) I5-10300H CPU @ 2.50ghz, RAM:8.00 GB, and NVIDIA GTX1660Ti. The programming language is Python3.7.0, and the compiler is PyCharm 2018 3.5 × 64. Anaconda4.5.11 is the basic platform for deep learning training. TensorFlow 1.14.0 and keras2.1.0 are deep learning frameworks.

### Data collection and preprocessing

The air quality data used in this experiment are obtained from http://data.epmap.org/. There are often some problems in the original data, such as missing and duplicating some data. Some of the original data of the summarized air quality data are shown in Table 2.

**Table 2:** Part of the original data.

Date	PM2.5 (μg/m^3^)	CO (μg/m^3^)	O_3_ (μg/m^3^)	NO_2_ (μg/m^3^)	PM10 (μg/m^3^)	SO_2_ (μg/m^3^)	AQI
2021/2/1 0:00:00	110	1.2	16	50	161	10	144
2021/2/1 1:00:00							
2021/2/1 2:00:00	105	1.2	8	52	160	8	138
2021/2/1 3:00:00	106	1.2	6	55	162	10	139
2021/2/1 3:21:00	106	1.2	6	55	162	10	139
2021/2/1 4:00:00	109	1.4	7	53	159	11	143

In this experiment, the original data are processed as follows:

1. Delete duplicates. There are duplicate data in the original data, for example, there are duplicate data in the data at 3:00 on February 1, 2021 and 3:21 on February 1, 2021. In this experiment, keep the last data and delete the previous duplicate data.
2. Data filling. In the process of air quality data detection, data loss may be caused by network interruption, storage failure, and other reasons, such as the data at 1:00 on February 1, 2021. These low-quality data will affect the model's learning effect. As a result, the final prediction accuracy is not high, and there is a problem of missing values in the original data. Considering that the air pollution data changes smoothly with time in most cases, and there is generally no sudden change in values, this experiment uses the average value of the data of one hour before and one hour after to fill in the missing parts^47^, as shown in formula (23).
  23 $${\mathrm{V}}_{\mathrm{t}}=\frac{{\mathrm{V}}_{\mathrm{t}-1}+{\mathrm{V}}_{\mathrm{t}+1}}{2},$$
  where ${\mathrm{V}}_{\mathrm{t}-1}$ is the data of one hour before time t, ${\mathrm{V}}_{\mathrm{t}}$ is the missing value at time t, and ${\mathrm{V}}_{\mathrm{t}+1}$ is the data of one hour after time t.

Because the environmental protection department calculates AQI through six main pollution indexes: PM2.5, CO, O_3_, NO_2_, PM10, and SO_2_, these six indexes are introduced as input items of the data set in this experiment^48–50^. Air quality data from 00:00 on April 4, 2019 to 23:00 on June 30, 2021 in Shijiazhuang city, Hebei Province, China are used as experimental data set. There are 39,408 pieces of data in this data set. The data obtained after data preprocessing in Table 2 are shown in Table 3.

**Table 3:** Experimental data.

Date	PM2.5 (μg/m^3^)	CO (μg/m^3^)	O_3_ (μg/m^3^)	NO_2_ (μg/m^3^)	PM10 (μg/m^3^)	SO_2_ (μg/m^3^)	AQI
2021/2/1 0:00:00	110	1.2	16	50	161	10	144
2021/2/1 1:00:00	107.5	1.2	12	51	160.5	9	141
2021/2/1 2:00:00	105	1.2	8	52	160	8	138
2021/2/1 3:21:00	106	1.2	6	55	162	10	139
2021/2/1 4:00:00	109	1.4	7	53	159	11	143

### Data normalization

There is a big difference between the sample values of some features and those of other features in the data set, which may lead to slow convergence and reduce the training accuracy of the model. In this experiment, z-score normalization processes the original data, as shown in formula (24), where $\upsigma$ is the standard deviation of the original data, $\overline{\mathrm{x} }$ is the mean of the original data, and ${\mathrm{x}}^{*}$ is the value after standardization. After the data standardization, the data is dimensionless and scaled to the same interval. In addition, the features are comparable, and the trend and relative size of the scaled data do not change, which speeds up the model convergence.

24 $${\mathrm{x}}^{*}=\frac{\mathrm{x}-\overline{\mathrm{x}}}{\sigma  }.$$

### Three-dimensional time series data construction

**Figure 11:**
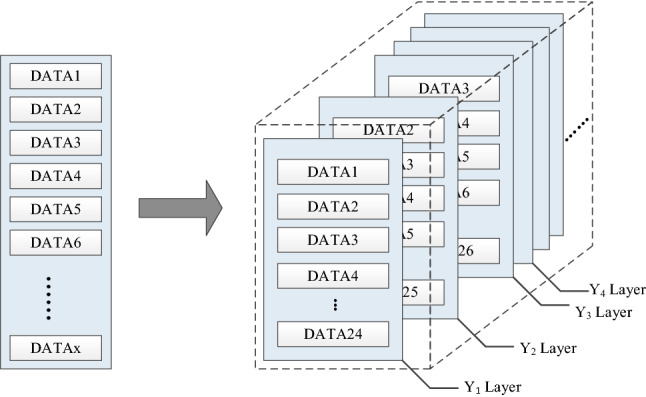
The construction process of three-dimensional time series.

This experiment uses the method of constructing time series, takes the time of the input data as a sequence, and carries out two-dimensional segmentation and three-dimensional construction of the input data. In Fig. 11, assuming that there are X pieces of experimental data, the data is constructed in three dimensions according to the setting of step = 1 and sequence = 24. The data from the first to the 24th constitute layer Y_1_, and data from the second to the 25th constitute layer Y_2_, and so on. Complete a total of X-23 layers (Y_1_, Y_2_… Y_X-32_) construction; each layer contains 24 pieces of data, that is, the three-dimensional data construction is completed. The constructed time series data are divided into training set, validation set, and test set in this experiment. The prediction model takes the first 23 data of each layer as input and the AQI value of the 24th layer as output for training, validation and evaluation.

### Data set segmentation

During the model designing and training process, model parameters (such as changing weights, choosing the number of layers and the size of each layer) need to be adjusted^51^. In the process of model training, it is necessary to provide feedback information through the prediction performance of the validation set, to adjust the network model and parameters, which is also the role of the validation set. However, in the training process, the information of the validation set will be leaked. The more feedback adjustment of the model, the more information will be leaked, so the model will more clearly "understand" the experimental set, which will eventually cause the model to fail on over-fitting on the validation set. At this time, a data set which is completely "unfamiliar" to the model—the test set is needed to measure the overall performance of the model prediction.

So after presetting model parameters, the data set will be divided into the training set, validation set, and test set. According to experience, the data volume ratios are as follows: 8:1:1, 7:2:1, 6:3:1, 7:1:2, 6:2:2, 5:3:2, 6:1:3, 5:2:3 and 4:3:3. In different data set segmentation ratios and different model prediction results, the prediction fitting degree of the validation set is shown in Table 4. In this experiment, when the data ratio of the training set, validation set and test set is 7:2:1, the prediction fitting degree of different models is higher. Therefore, the ratio of the training set, validation set and test set in this experiment is 7:2:1.

**Table 4:** Fitting degree of different prediction models in segmentation of data sets with different ratios.

Model	8:1:1	7:2:1	6:3:1	7:1:2	6:2:2	5:3:2	6:1:3	5:2:3	4:3:3
	R^2^	R^2^	R^2^	R^2^	R^2^	R^2^	R^2^	R^2^	R^2^
SVR	0.8852	0.8762	0.8633	0.8232	0.8269	0.8132	0.7795	0.7487	0.7516
RFR	0.9001	0.8969	0.8425	0.8356	0.8125	0.8019	0.7851	0.7421	0.7359
MLP	0.8932	0.9061	0.8821	0.8692	0.8793	0.8611	0.7932	0.7752	0.8003
LSTM	0.9355	0.9365	0.9210	0.8716	0.8921	0.8862	0.8856	0.8236	0.8526
GRU	0.9492	0.9507	0.9315	0.8796	0.8880	0.8760	0.8569	0.8525	0.8210
ILSTM	0.9420	0.9470	0.9399	0.8890	0.8965	0.8890	0.8611	0.8499	0.8511
CNN-LSTM	0.9410	0.9487	0.9280	0.9001	0.9034	0.8962	0.8960	0.8321	0.8561
CNN-GRU	0.9498	0.9512	0.9392	0.9030	0.9164	0.8836	0.8695	0.8530	0.8312
CNN-ILSTM	0.9510	0.9638	0.9330	0.9068	0.9186	0.9020	0.8741	0.8499	0.8501

Because the neural network has a strong fitting ability, if the data set is trained in chronological order and the "batch" of the same combination appears repeatedly, the model may produce an over-fitting state through learning, thus affecting the test of the generalization ability of the model in the experiment. Therefore, in the process of this experiment, the order of data input is interrupted in every training, validation, and test of the model.

### Model parameter adjustment

In deep learning, a given machine learning algorithm has model parameters and model hyper-parameters. Model parameters are generally internal variables, such as bias, weight, etc. These parameters are not set manually but are automatically learned and obtained through model training data. The model's hyper-parameters are set before the model training and are often designed manually by the experience of researchers. Model hyper-parameters can be divided into structural hyper-parameters and running hyper-parameters. Structural hyper-parameters refer to configurations that play a decisive role in model structure, such as filters, padding, and kernel_size in convolution layer; pool_size and padding in pooling layer; units and kernel_initializer in ILSTM layer. Running hyper-parameters are used to run neural networks, such as batch_size, epochs, and learning_rate. Traditional manual design of hyper-parameters is time-consuming, inefficient, and costly, and even the results of the hyper-parameters model designed by experimenters are difficult to reproduce and expand. This experiment combines empirical mode and hyper-parameter optimization technology (Grid search optimization algorithm) to adjust parameters. The purpose of hyper-parameter optimization is to find a suitable set of parameters in the algorithm model so that the model has good expression ability and generalization ability. Based on experience, we select the parameters of batch_size using 110, 120, 130, 140, and 150. We select epochs using 80, 90, 100, and 110. We select learn_rate using 0.01, 0.005, 0.002, 0.001, and 0.0009. After selecting filters, pool_size, units, learning_rate, and other parameters by grid search optimization algorithm, the details of the parameters set in the final experiment are shown in Table 5.

**Table 5:** Model parameter.

Layer	Parameter
CNN parameter	Filters = 16; kernel_size = 1; padding = ‘valid’
MaxPooling1D parameter	Pool_size = 1; padding = ‘valid’
ILSTM parameter	Units = 16; kernel_initializer = ‘he_normal’
Other parameter	Loss = ‘mae’; batch_size = 130; learning_rate = 0.001; epochs = 100

### Experiment analysis

#### Model convergence

**Figure 12:**
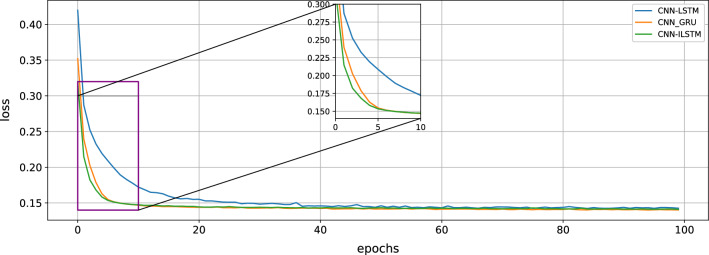
Convergence of CNN-LSTM, CNN-GRU and CNN-ILSTM.

After the model is built and the parameters are set, it is necessary to verify whether CNN-ILSTM normally converges during training. When all parameters are the same and epoch = 100, 1–0 loss function is used in this experiment to show its convergence. In this experiment, the convergences of CNN-ILSTM, CNN-GRU, and CNN-LSTM are shown in Fig. 12. The loss function of CNN-ILSTM is smaller than that of CNN-LSTM before training 10 times, so the convergence speed of CNN-ILSTM is faster than that of CNN-LSTM in this experiment. The loss function of CNN-ILSTM is smaller than that of CNN-GRU before training 5 times, so the convergence speed of CNN-ILSTM is faster than that of CNN-GRU in this experiment.

#### Evaluation index

To scientifically evaluate the prediction accuracy of the model, this experiment uses MAE, MSE, R^2^, and model training time as the overall evaluation index of the model. MAE describes how different the predicted value is from the true value. MSE measures the average modulus length of the predicted value error, regardless of direction. R^2^ describes how similar the predicted value is to the true value. The model training time describes the calculation time of the model. The calculation methods of MAE, MSE and R^2^ are shown in formula (25–27).

25 $$ {\text{MAE}} = \frac{1}{{\text{m}}}\mathop \sum \limits_{{{\text{i}} = 1}}^{{\text{m}}} \left| {\left( {{\text{y}}_{{\text{i}}} - {\hat{\text{y}}}_{{\text{i}}} } \right)} \right|, $$

26 $$ {\text{MSE}} = \frac{1}{{\text{m}}}\mathop \sum \limits_{{{\text{i}} = 1}}^{{\text{m}}} \left( {{\text{y}}_{{\text{i}}} - {\hat{\text{y}}}_{{\text{i}}} } \right)^{2} , $$

27 $$ {\text{R}}^{2} = 1 - \frac{{\mathop \sum \nolimits_{{\text{i}}} \left( {{\hat{\text{y}}}_{{\text{i}}} - {\text{y}}_{{\text{i}}} } \right)^{2} }}{{\mathop \sum \nolimits_{{\text{i}}} \left( {{\overline{\text{y}}}_{{\text{i}}} - {\text{y}}_{{\text{i}}} } \right)^{2} }}, $$

where m is the number of data in the test set; ${\hat{\text{y}}}_{{\text{i}}}$ is the predicted value; ${\mathrm{y}}_{\mathrm{i}}$ is the true value; ${\overline{\mathrm{y}} }_{\mathrm{i}}$ is the average value of the true values.

#### Experimental results

To verify the accuracy of CNN-ILSTM in predicting AQI, traditional regression models (SVR, RFR, and MLP), recurrent neural network models based on gated technology (LSTM, GRU, ILSTM), and hybrid prediction models (CNN-LSTM, CNN-GRU) are introduced as comparison models.

**Figure 13:**
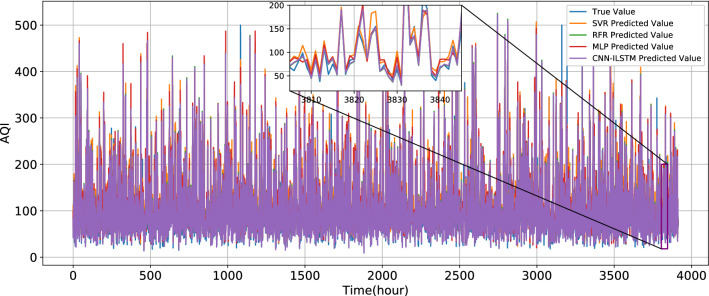
True value and the predicted value of SVR, RFR, MLP, and CNN-ILSTM.

Experimental results are shown in Table 6. The test set prediction evaluation results show that the traditional regression models SVR, RFR, and MLP have a lower prediction fitting degree than the recurrent neural network model based on gated technology. The R^2^ of LSTM is 0.0697 higher than that of SVR, the R^2^ of LSTM is 0.0542 higher than that of RFR, and the R^2^ of LSTM is 0.0341 higher than that of MLP. The predicted and true values of SVR, RFR, MLP, and CNN-ILSTM are shown in Fig. 13.

**Table 6:** Experimental results.

Model	MAE	MSE	R^2^	Training time (s)
SVR	19.2644	483.6269	0.8750	411.2
RFR	15.364	402.6292	0.8905	341.3
MLP	13.6186	386.3648	0.9106	50.3
LSTM	9.4974	266.8523	0.9447	124.9
GRU	11.0032	270.8179	0.9411	71.6
ILSTM	9.2420	248.9234	0.9508	64.2
CNN-LSTM	9.2314	258.5143	0.9466	149.5
CNN-GRU	9.0615	248.6389	0.9478	92.5
CNN-ILSTM	8.4134	202.1923	0.9601	85.3

**Figure 14:**
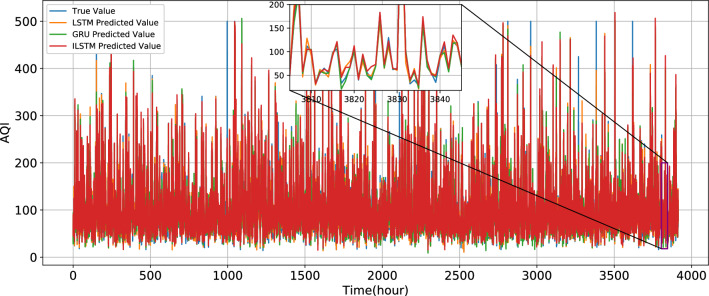
True value and the predict value of LSTM, GRU, and ILSTM.

**Figure 15:**
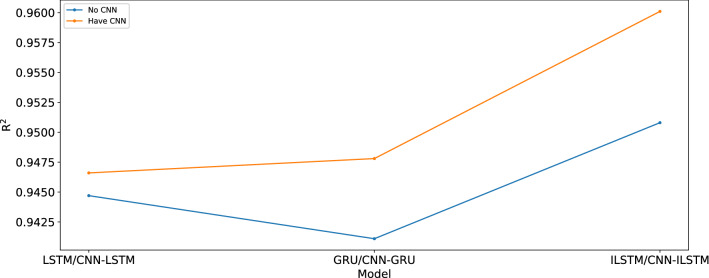
Comparison of ILSTM, LSTM and GRU with or without CNN.

Compared with LSTM and GRU, R^2^ of ILSTM increases by 0.0061 and 0.0097, respectively. MAE decreases by 0.2554 and 1.7612, respectively. The true value and predicted values of LSTM, GRU, and ILSTM are shown in Fig. 14. As shown in Fig. 15, in this experiment, the different prediction models have improved their fitting degree after the introduction of CNN. Compared with LSTM, R^2^ of CNN-LSTM increases by 0.0019, MAE decreases by 0.266, and MSE decreases by 8.338. Compared with GRU, R^2^ of CNN-GRU increases by 0.0067, MAE decreases by 1.9417, and MSE decreases by 22.179. Compared with ILSTM, R^2^ of CNN-ILSTM increases by 0.0093, MAE decreases by 0.8286, and MSE decreases by 46.7311.

**Figure 16:**
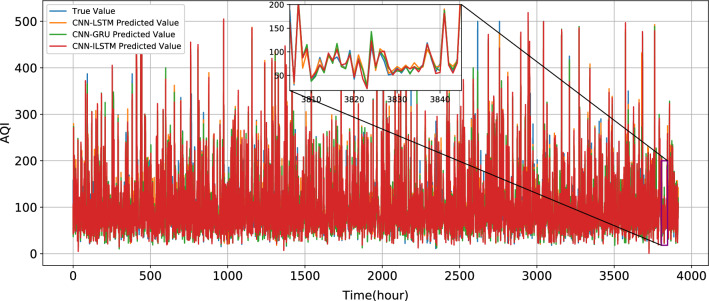
True value and the predict value of CNN-LSTM, CNN-GRU, and CNN-ILSTM.

And in the prediction of this test set, CNN-ILSTM prediction evaluation is the best. Compared with CNN-LSTM, R^2^ of CNN-ILSTM increases by 0.0135, MAE decreases by 0.818, and MSE decreases by 56.3220. Compared with CNN-GRU, R^2^ of CNN-ILSTM increases by 0.0123, MAE decreases by 0.6481, and MSE decreases by 46.4466. In terms of training time, SVR and RFR take a high time, reaching 411.2 s and 341.3 s respectively. Compared with LSTM, the training time of ILSTM decreases by 48.60%. Compared with GRU, the training time of ILSTM decreases by 10.34%. Compared with CNN-LSTM, the training time of CNN-ILSTM decreases by 42.94%. Compared with CNN-GRU, the training time of CNN-ILSTM decreases by 7.78%. The true value and predicted values of CNN-LSTM, CNN- GRU, and CNN-ILSTM are shown in Fig. 16.

#### Discussion

In the experiment using this test set, the overall evaluation index of the CNN-ILSTM AQI prediction model performs better than other models. Compared with the traditional regression models, the recurrent neural network models based on the gated technology have a better prediction fitting degree. Compared with LSTM and GRU, ILSTM significantly reduces the training time due to the reduction of ILSTM parameters on the premise of maintaining higher prediction accuracy. CNN-ILSTM compared with ILSTM, the introduction of CNN improves the prediction accuracy. Compared with CNN-LSTM and CNN-GRU, the prediction accuracy and training time of CNN-ILSTM are better.

The reasons for the improvement of CNN-ILSTM's AQI prediction accuracy are as follows:

1. The introduction of the model makes up for the shortcomings of the single model in some aspects. For example, CNN can realize the advantages of data feature extraction, which makes up for the RNN’s deficiencies in eigenvalues screening and learning before data input.
2. ILSTM deletes the output gate, improves the input gate and forget gate, and introduces a CIM to learn historical data more thoroughly.

ILSTM and CNN-ILSTM have been greatly improved in model training time because:

1. The structure of the ILSTM model is simpler. ILSTM consists of input gate and forget gate. Compared with LSTM, ILSTM has no output gate.
2. ILSTM has fewer parameters. Compared with LSTM, ILSTM reduces weight parameters from 8 to 4 and bias parameters from 4 to 2. Compared with GRU, ILSTM reduces weight parameters from 6 to 4 and bias parameters from 3 to 2.

## Conclusions

This paper presents an AQI prediction model based on CNN-ILSTM. Compared with the traditional regression models of SVR, RFR, and MLP, and the deep learning models of LSTM, GRU, ILSTM, CNN-LSTM, and CNN-GRU, the overall evaluation of prediction results of CNN-ILSTM is best. ILSTM is proposed for the first time. ILSTM is improved and optimized in model design and parameter ratio on the premise of high prediction accuracy and alleviating the issues of “gradient explosion” and “gradient disappearance” in the RNN caused by long-term data dependence. Compared with LSTM and GRU, the training time of ILSTM is reduced by 48.6% and 10.34%, and ILSTM has the best AQI prediction results. In addition, the introduction of CNN makes up for the deficiency of ILSTM feature extraction and learning. The experiment results show that the MAE of CNN-ILSTM decreases by 0.284798, and the R^2^ increases by 0.013951 compared with ILSTM AQI prediction. The conclusions of this paper are as follows:

1. ILSTM has performed better than LSTM in my tests. ILSTM is an improvement of LSTM, which deletes the output gate in LSTM, improves its input gate and forget gate, and introduces a CIM to prevent supersaturation in the learning process. On the premise of ensuring that the model can alleviate the issues of “gradient explosion” and “gradient disappearance” of RNN and has high prediction accuracy. Compared with LSTM and GRU, ILSTM significantly reduces the training time.
2. The AQI prediction model of CNN-ILSTM makes up for the shortcomings of the single prediction model, such as insufficient feature data extraction and insufficient historical data learning. In this experiment, the AQI prediction model of CNN-ILSTM is the best.
3. The model design and parameter tuning are improved and optimized, so the convergence rate of the AQI prediction model based on CNN-ILSTM is improved.

However, the AQI prediction model of CNN-ILSTM does not perform well in extreme value prediction. Therefore, the following research will carry out the high-precision prediction of extreme values.
